# Microangiopathic Hemolytic Anemia and Fulminant Renal Failure: A Rare Manifestation of Pheochromocytoma

**DOI:** 10.1155/2019/2397638

**Published:** 2019-12-14

**Authors:** Nishant Sharma, Divya Ravi, Mehvish Khan, Metlapalli Venkata Sravanthi, Mark M. Aloysius

**Affiliations:** The Wright Center for Graduate Medical Education, 111 N Washington Ave, Scranton, PA 18503, USA

## Abstract

Pheochromocytoma is a rare adrenal tumor that is classically associated with the triad of paroxysmal tachycardia, diaphoresis, and headaches. However, it can have myriad manifestations. We present a case of a 31-year-old male who presented with abdominal pain, hypertensive emergency, and renal failure. Abdominal imaging demonstrated a left adrenal mass. Plasma metanephrines (153 pg/ml, *n* < 57) and normetanephrines (1197 pg/ml, *n* < 148) were noted to be elevated, leading to the diagnosis of pheochromocytoma. Intravenous antihypertensives were utilized to control his blood pressure. Hemodialysis was initiated given the degree of renal dysfunction. The patient subsequently developed hemolytic anemia, requiring the transfusion of multiple units of packed red cells. He developed acute respiratory failure leading to intubation, but was thereafter liberated from the ventilator following clinical stabilization. Uncontrolled hypertension precipitated by pheochromocytoma can cause microangiopathic hemolytic anemia and renal insufficiency. This case is notable not only for the occurrence of this rare presentation, but also for the severity of manifestations in a young male with no known significant comorbidities.

## 1. Introduction

Pheochromocytoma is a rare form of catecholamine-secreting adrenal tumor with an estimated annual incidence of about three to eight per million [1]. About 30 percent of pheochromocytoma occur in association with familial neuroendocrine syndromes, while a majority are sporadic. Pheochromocytoma typically presents in the fourth or fifth decade of life, with no specific gender predilection [2]. Historically, pheochromocytoma has been outlined by the classic triad of paroxysmal tachycardia, diaphoresis, and headaches in the setting of uncontrolled hypertension. However, most patients do not manifest with these typical symptoms; pheochromocytoma has been labelled “the great mimic” [3] that can rarely present with dramatic multiorgan crisis or a “pheochromocytoma crisis”, as described in our case below.

## 2. Case Presentation

The patient is a 31-year-old African American male who presented to the ED with a chief complaint of upper abdominal pain. This abdominal pain had begun two weeks prior and was progressively worsening. He described it as a mid-epigastric dull ache, aggravated by eating, and lacking in radiation or alleviating factors. He reported associated nausea, vomiting, and nonbloody diarrhea, as well as a weight loss of 8 pounds over the same period of time, which is something he attributed to poor oral intake. A complete review of systems was limited as the patient was lethargic and slow to answer questions. His medical history was limited to an episode of pancreatitis about 5 years prior to presentation. He had not seen a doctor for years prior to presentation, but was told that he had high blood pressure at a plasma donation center a few years before. He took no medications apart from over the counter ibuprofen as needed for pain. His family history was significant only for hypertension in his mother. He had a 10-pack year smoking history, and biweekly use of marijuana. He denied consumption of alcohol and other illicit drugs.

**Figure 1:**
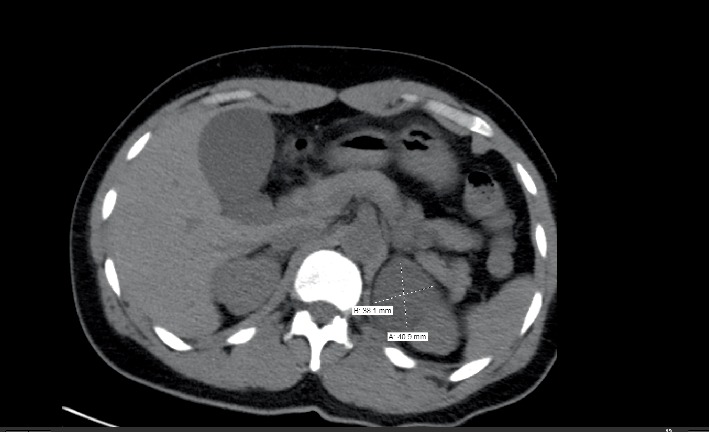
CT abdomen demonstrating adrenal tumor.

At presentation, he was found to have a blood pressure of 223/142 mm Hg. Physical exam was notable for moderate epigastric tenderness. His lab work was most notable for leukocytosis (12.2 × 109/*μ*L, *n* < 10), normocytic anemia (Hb 7.8 g/dL, *n* = 14 − 17), thrombocytopenia (100 × 109/L, *n* = 150 − 450), deranged renal function (BUN 124 mg/dL, *n* < 20, creatinine 21.6 mg/dL, *n* < 1.3), proteinuria (≥500 mg/dL, *n* < 20), mild hematuria with metabolic acidosis (pH of 7.25), and anion (gap of 20). A CT scan of the abdomen demonstrated acute colitis, and a low density left adrenal mass measuring 4.4 × 4.4 × 4.5 cm (Figure 1).

**Figure 2:**
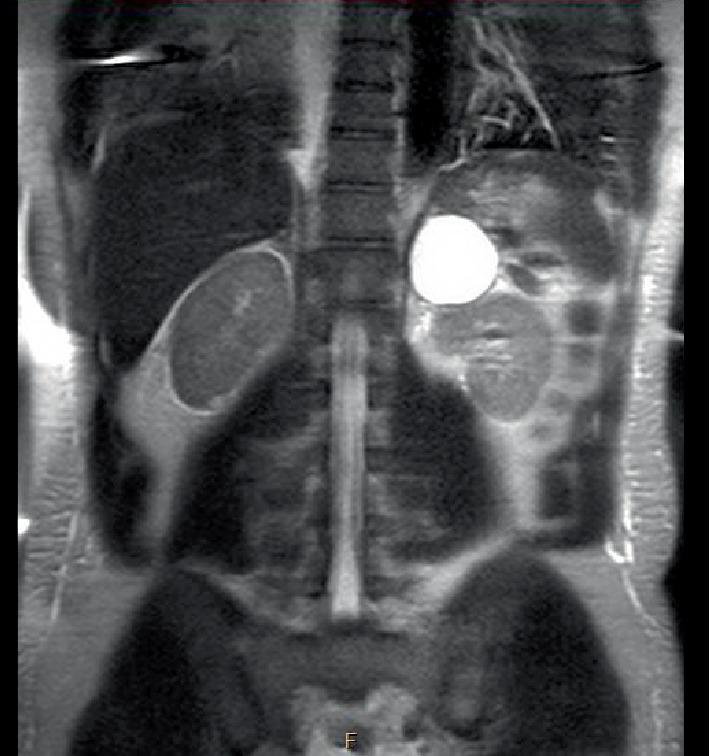
MRI abdomen demonstrating left adrenal mass.

The patient was admitted to the intensive care unit; nicardipine drip was initiated to lower the patient's blood pressure and broad-spectrum antibiotic coverage was started for ongoing colitis. A comprehensive workup for secondary causes of hypertension was ordered, including plasma metanephrines, plasma normetanephrines, plasma renin, plasma angiotensin, a 24-hour urine cortisol, thyroid function tests, and a renal ultrasound. A tunnelled catheter was placed for urgent initiation of dialysis given his severe degree of uremia and possible encephalopathy. The next day, he developed worsening anemia necessitating the transfusion of two units of packed red cells. Additionally, he had an elevated LDH, undetectable haptoglobin, elevated reticulocyte count, negative Coomb's test, and thrombocytopenia. A peripheral smear was notable for the presence of schistocytes. He was transitioned off the nicardipine infusion to oral amlodipine and labetalol. An MRI of the abdomen assisted in confirming and further characterizing the adrenal lesion (Figure 2).

The following day, during a session of hemodialysis, the patient developed acute respiratory distress requiring intubation for airway support and resumption of nicardipine infusion for hypertensive emergency. A chest X-ray demonstrated bilateral alveolar infiltrates and pleural effusion. High sensitivity troponin was found to be elevated and a transthoracic echocardiogram showed mildly impaired left ventricular function with an ejection fraction of 50% and no significant valvular abnormalities. Over the next 48 hours, his oxygen needs improved and he was subsequently extubated. The workup for secondary hypertension revealed elevated plasma metanephrines (153 pg/ml, *n* < 57) and normetanephrines (1197 pg/ml, *n* < 148), thus confirming the diagnosis of pheochromocytoma presenting as hypertensive emergency. Hemodialysis was resumed and he was started on the appropriate antihypertensive medications. Once deemed clinically stable, the patient was transferred to a tertiary care center for evaluation of surgical resection of the tumor.

## 3. Discussion

Pheochromocytoma is a rare catecholamine secreting tumor, occurring in less than 0.5% of patients with hypertension [4], which classically presents with hypertension, headache, palpitations, sweating, and diaphoresis. It can, however, rarely have more dramatic presentations, including cardiogenic shock [5, 6], myocardial infarction [7], cardiomyopathy [8], rhabdomyolysis [9–12], acute renal failure [5, 13–16], pulmonary haemorrhage [7], arrhythmia, and microangiopathic hemolysis [13, 14, 17]. An acute and severe presentation of pheochromocytoma involving end organ damage or dysfunction brought on by catecholamine-induced hemodynamic instability is referred to as a pheochromocytoma crisis (PCC). While there is a wide variation in mortality estimates, a recent review estimates it to be 15% [18]. One proposed classification system describes two types of crises-Type A, characterized by hemodynamic instability and end organ damage of one or more organ systems, and Type B, characterized by sustained hypotension, or shock, and multiorgan system damage [18]. This case of PCC was notable not only for the presence of two rare manifestations of pheochromocytoma—microangiopathic hemolytic anemia and renal failure—but also for their severity. The patient had severe renal insufficiency with a serum creatinine of 21.6 and estimated GFR of 3 mL/min per 1.73 m^2^, and a degree of hemolysis that produced a precipitous drop in hemoglobin.

There are multiple possible mechanisms of renal insufficiency in a patient with pheochromocytoma. There have been several case reports detailing the occurrence of acute renal failure as a consequence catecholamine induced vasoconstriction [9, 10]. Another mechanism for renal dysfunction in the setting of pheochromocytoma is hypertension induced intimal thickening of glomerular vasculature, and glomerulosclerosis. It is also important to note the concentric left ventricular hypertrophy on ECHO and the EKG, which is a manifestation of sustained uncontrolled hypertension. Given these findings and lack of medical visits since his remote presentation of elevated BP in a blood donation center few years prior, we propose that this patient's severe renal insufficiency was the result of hypertensive nephropathy possibly complicated by rhabdomyolysis. A renal biopsy, which could have confirmed this hypothesis, was deemed high risk in this patient given the atrophic nature of his kidneys. The co-occurence of rhabdomyolysis as a consequence of catecholamine induced vasoconstriction and resultant muscle ischemia might have made a minor contribution to this patient's renal insufficiency; urinalysis did demonstrate a positive result for blood and only a few RBC on microscopy. A creatine kinase level was not obtained as a part of initial work up; this is one limitation of this case report. Due to the degree of uremia, a tunnelled catheter was placed on an emergent basis for the initiation of dialysis.

Another distinguishing feature of this case is the severe degree of hemolytic anemia, which can be attributed to one or more of the following mechanisms. Uncontrolled hypertension, in itself has been shown to cause microangiopathic hemolytic anemia (MAHA) and thrombocytopenia due to endothelial injury and subsequent destruction of RBCs and platelets [19]. The subnormal haptoglobin coupled with elevated LDH and reticulocyte count, and schistocytes on peripheral smear favor MAHA. There is also some evidence to suggest the role of elevated plasma renin activity (PRA), as noted in this case, in causing accelerated vascular damage and renal dysfunction resulting in microangiopathic hemolysis [20]. Lastly, a high normal vitamin B12 level, negative complement panel and negative Coombs test make other mechanisms of hemolysis less likely.

A review of literature shows us several cases of reported renal failure in the setting of pheochromocytoma secondary to rhabdomyolysis [9, 10] or cardiogenic shock [5]. The concurrence of pheochromocytoma, malignant hypertension, renal failure and microangiopathic hemolytic anemia appears to a rare presentation, with only two reported cases found in our literature review [13, 14]. Our case adds to the body of literature by demonstrating the extent of severity to which pheochromocytoma can cause hematological and renal insults through a combination of mechanisms in a young patient with no significant comorbidities. Schweizer et al. suggest that plasmapheresis can lead to favorable outcomes in pheochromocytoma with thrombotic microangiopathy [14]. However, since the ADAMTS13 activity in our patient was found to be normal, we suggest that the optimum management of both renal failure and microangiopathic hemolytic anemia in a case like ours involves prompt control of hemodynamics, renal replacement therapy, and treatment directed towards the adrenal tumor itself.

A final note must be made on the diagnostic utility of plasma metanephrine and normetanephrine in pheochromocytoma. The Endocrine Society (ES), in their clinical practice guidelines for pheochromocytoma and paraganglioma (PPGL) [21] recommend the measurement of plasma free metanephrines or urinary fractionated metanephrines in the initial biochemical testing for PPGL. However, being metabolites of catecholamines, their levels in the plasma can be elevated in conditions of physiological stress, such as an acute illness [22]. They can be elevated in up to 75% of patients with acute cardiomyopathy [23]. Additionally, renal dysfunction itself may contribute to the elevation of these metabolites [24]. The aforementioned ES guidelines reports a low probability of false positivity in case with elevations of both normetanephrine and metanephrine, or an isolated elevation 3-fold or more above upper cutoffs in either metabolite. In this case, both metanephrine and normetanephrine were found to be elevated, with the latter being 8 fold the upper limit of normal. The coincident identification of an adrenal mass, and characteristic symptoms imparts a high probability for the diagnosis of pheochromocytoma in this patient. A clonidine suppression test may be employed for the differentiation of true-positive from false-positive borderline elevation of normetanephrine [21].

Pheochromocytoma is a disease with a myriad of possible presentations. In a previously healthy young patient, presenting with a triad of malignant hypertension, hemolytic anemia and renal failure, pheochromocytoma must be considered as part of the differential.
